# An easy operating pathogen microarray (EOPM) platform for rapid screening of vertebrate pathogens

**DOI:** 10.1186/1471-2334-13-437

**Published:** 2013-09-20

**Authors:** Weiwei Huang, Yinhui Yang, Xinlei Zhang, Changan Zhao, Aihua Yin, Xiaozhuang Zhang, Zhengxin He, Yongqiang Jiang, Liang Zhang

**Affiliations:** 1 BioChain (Beijing) Science & Technology Inc., No.7A, Yongchang North Rd, Business Development Area, Beijing 100176, China; 2 State Key Laboratory of Pathogens and Biosecurity, Beijing Institute of Microbiology and Epidemiology, Beijing 100071, China; 3 Beijing Genestone Biocomputing Corporation, Beijing 100101, China; 4 Guangdong Women and Children’s Hospital, Guangzhou, Guangdong 510010, China; 5 Department of Clinical Laboratory, Bethune International Peace Hospital of PLA, Shijiazhuang, Hebei 050082, China

## Abstract

**Background:**

Infectious diseases emerge frequently in China, partly because of its large and highly mobile population. Therefore, a rapid and cost-effective pathogen screening method with broad coverage is required for prevention and control of infectious diseases. The availability of a large number of microbial genome sequences generated by conventional Sanger sequencing and next generation sequencing has enabled the development of a high-throughput high-density microarray platform for rapid large-scale screening of vertebrate pathogens.

**Methods:**

An easy operating pathogen microarray (EOPM) was designed to detect almost all known pathogens and related species based on their genomic sequences. For effective identification of pathogens from EOPM data, a statistical enrichment algorithm has been proposed, and further implemented in a user-friendly web-based interface.

**Results:**

Using multiple probes designed to specifically detect a microbial genus or species, EOPM can correctly identify known pathogens at the species or genus level in blinded testing. Despite a lower sensitivity than PCR, EOPM is sufficiently sensitive to detect the predominant pathogens causing clinical symptoms. During application in two recent clinical infectious disease outbreaks in China, EOPM successfully identified the responsible pathogens.

**Conclusions:**

EOPM is an effective surveillance platform for infectious diseases, and can play an important role in infectious disease control.

## Background

The frequent invasion of microorganisms, including viruses, bacteria, fungi, parasites, and other eukaryotic and prokaryotic organisms, has threatened and will continue to threaten the life and health of humans and other vertebrates. In recent years, mutant or new forms of some existing pathogens have been identified as the causative agents of a number of outbreaks that have endangered public health in China [1]. Severe acute respiratory syndrome (SARS), caused by a coronavirus, spread throughout Guangdong Province in 2003, followed by a worldwide epidemic. During the epidemic, 66% of the SARS cases were reported in China, resulting in 349 human deaths [2]. In 2007, an outbreak of hand, foot, and mouth disease (HFMD) infected 1149 persons and caused the death of three children in Linyi City, Shandong Province, China [3]. The 2009 influenza A (H1N1) pandemic affected more than 154,000 human patients, leading to 842 deaths in China alone [4]. Because of its large and highly mobile population, the emergence of infectious diseases in China is relatively more frequent. Therefore, a system implemented by the medical community and government for the monitoring of pathogens that could have a significantly negative impact on public health is urgently required in China.

China has an established hospital-based surveillance system for infectious diseases. All clinical and hospital reports of both suspected and confirmed cases of notifiable infectious disease must be sent to local Centers for Disease Control (CDC). The information is then sent to the China CDC headquarters in Beijing through the National Infectious Diseases Monitoring Information System Database, which was established in 2004. The hierarchical administrative organization of the surveillance system ensures a rapid and efficient upward flow of epidemic information [5]. Based on this system, development of effective diagnostic platforms can greatly enhance the prevention and control of infectious diseases in China. The predominant techniques for identification of microbial pathogens depend on conventional clinical microbiology monitoring approaches. Although well established, these approaches usually require culture of the pathogens, followed by susceptibility tests, which are time-consuming and laborious. In addition, many microbes are difficult to culture, and may be undetectable by culture-based approaches.

Molecular approaches for microbial surveillance and discovery have emerged as a very promising alternative for early diagnosis of infectious diseases. Currently, molecular approaches include traditional Sanger DNA sequencing, polymerase chain reaction (PCR), oligonucleotide microarrays, and next generation sequencing (NGS). Among these four technologies, the former two can identify a few known pathogens that must then be confirmed individually, and thus cannot cover a wide range of pathogens. The latter two methods cover a broad range of pathogens, and are therefore suitable for identifying unknown or even novel pathogens in infectious outbreaks. Although NGS produces the most in-depth, unbiased information, and can reveal completely novel organisms, it is time-consuming and expensive, especially for the analysis of complex samples [6]. DeRisi and colleagues developed the first generation of microarray platform, called ViroChip, to detect a wide range of viruses [7]. In 2003, the ViroChip helped to characterize SARS as a novel *Coronavirus*[8]. Since then, ViroChip has also been used to detected a human metapneumovirus [9], a novel influenza virus [10], and a novel adenovirus [11]. More recently, GreeneChip and MDA microarrays have been developed, which are broader spectrum approaches that can detect several thousand pathogenic viruses, bacteria, fungi, and protozoa [12,13]. The aforementioned three platforms all used long oligonucleotide probes and random amplification of nucleic acids.

In this study, we report the construction of a high throughput pathogen microarray platform, named Easy Operating Pathogen Microarray (EOPM), for large-scale pathogen surveillance and discovery in China. The platform uses similar technical features to previous methods, but will be more useful for clinical applications because of its user-friendly analysis software. The EOPM was designed based on the latest versions of nucleic acid sequence resources for microbes. Clinical application of the microarray system confirmed that it can correctly identify the pathogens responsible for infectious disease.

## Methods

### Collection of nucleic acid sequences of vertebrate pathogens

Release 111 of the European Molecular Biology Laboratory (EMBL, http://www.embl.org/) database (March 2012) was used to establish our vertebrate viral sequence database. The terms at the family level that describe the host as a vertebrate animal were extracted from the “Virus Taxonomy List 2012” (http://ictvonline.org/virusTaxonomy.asp?version=2012), compiled by the International Committee on Taxonomy of Viruses (ICTVdB). We only considered viruses under these taxonomy nodes. We also downloaded the sequences of fungi and parasites from EMBL. 18S rRNA sequences were extracted using the CDS tag. Finally, we obtained bacterial 16S rRNA sequences from the Ribosomal Database Project (RDP 10.28, http://rdp.cme.msu.edu). The final integrated dataset included 1,358,528 viral sequences representing complete and partial viral genomes, 2,110,258 bacterial 16S rRNA sequences, 621,351 fungal 18S rRNA sequences, and 1,735,744 18S rRNA sequences from parasites. The EOPM Chip distinguishes all 2,554 known vertebrate virus species (involving 151 genera, 36 families), 124 bacterial genera (involving 53 families), 38 fungal genera (involving 17 families), and 47 genera of parasites (involving 24 families). Considering that bacterial 16S rRNA genes show a relatively high level of homology, and that bacteria require the presence of active virulence genes for pathogenesis, 58 virulence genes were selected, including *rfbE*, *slt*-1, *ipaA*, and *katG*, and probes were designed against these gene sequences.

### EOPM chip design and fabrication

The basic design of the viral probes included as many different genomic target regions as possible for each species of vertebrate virus in the EMBLdB. First, probes were targeted to conserved regions in areas encoding the structural proteins. The protein families database (Pfam, http://pfam.sanger.ac.uk/) of multiple sequence alignments was used to cluster the functionally related sequences [14]. The regions tagged as 5′ UTR, 3′ UTR, and LTR were also extracted and used as candidate sequences for the following probe design. Second, candidate probes were screened according to the following criteria: probes with a length of 60 nt, no repeats exceeding a length of 8 nt, no hairpins with stem lengths exceeding 10 nt, GC content between 30–70%, and Tm from 60–80°C. Third, we used BLAST analysis to select the conserved viral probes at the genus level from all of the candidate probes. The extent of conservation was evaluated for each probe, and all were found to detect the majority of species in each genus. A target species was considered to be represented if a probe matched it with at least 75% sequence identity. Probes conserved at the genus level were selected based on a flexible threshold because the sequence conservation between species belonging to different genera is quite variable. Finally, we aligned the sequences of all the candidate probes against the nt database, which was downloaded from NCBI FTP in August 2012. Probes with high sequence similarity to non-target genomes were eliminated. Both species-specific and genus-conserved probes were included in the final probe set.

The identification of bacterial, fungal, and parasite probes was similar, but only focused on the 16S and 18S rRNA sequences. In addition, probes were also designed to target 1160 host immune response genes as a potential index to pathogenesis.

The 60-mer oligonucleotide probes were synthesized on a 75 mm × 25 mm glass slide by applying an inkjet deposition system (Agilent Technologies, Palo Alto, CA). A total of eight sub-arrays with 60,000 distinct 60-mer probes in one slide were customized. All hybridizations involved a fluorescently-labeled synthetic oligonucleotide that was complementary to a positive control probe, which was replicated for more than 4,000 spots scattered in different zones of each sub-array. This ensured that signals appeared in every zone of each sub-array to facilitate data extraction from hybridization figures.

### Sample preparation and EOPM hybridization

Microbial nucleic acids were extracted from serum, plasma, throat swabs, nasal lavage, feces, cerebrospinal fluid, and other body fluid using a TIANamp Virus DNA/RNA Kit (TIANGEN Biotech., Beijing, China). The carrier RNA from the kit was applied to extract virus nucleic acid with low molecular weight. The kit can be used to extract the nucleic acid from both RNA and DNA viruses (like adenovirus), as well as bacteria, fungi, and parasites. A previously described random PCR amplification strategy [7] with minor modification was applied to amplify extracted nucleic acids and label amplified products with fluorescent dye. In brief, the first cDNA strand was reverse transcribed with a random decamer heeled with a PCR primer (5′-GTTTCCCAGTCACGATCNNNNNNNNN-3′). The first strand cDNA was then synthesized to double-stranded DNA using the same primer and Klenow DNA polymerase (Takara, Dalian, China). Double stranded cDNA from both patients and normal controls was PCR amplified using the heel primer. Resultant PCR amplicons were then purified and labeled with Cy3-dCTP or Cy5-dCTP for the normal controls and patient samples, respectively, using Klenow polymerase (Takara). Labeled DNA was mixed with 60 μl of hybridization buffer and added to the 8 × 60,000 EOPM arrays for hybridization overnight at 65°C in a hybridization oven (Agilent). The EOPM arrays were then washed with 2× SSC, 0.005% Triton X-100 at room temperature for 1 min, followed by a second wash with 0.2× SSC at 37°C for 1 min. The arrays were then scanned using a dual-laser scanner (Agilent) and the images were extracted and analyzed using Feature Extraction software (Agilent).

### EOPM data analysis

The normal distribution of microbes in the human body should be considered when using EOPM to identify pathogens that are responsible for obvious clinical symptoms. We used two strategies to eliminate the background of normal microflora. Firstly, at the experimental level, we always compared the suspected clinical sample with a normal sample of the same type, i.e. serum vs. serum or feces vs. feces. Secondly, on a database level, we compared clinical samples with the same type of samples from a database that included more than 30 different samples from a normal population, such as serum, feces, cerebrospinal fluid, and throat swabs. The second aspect may avoid unexpected issues in the experimental normal control. Under the above strategy, each clinical sample was first compared with a normal control, and then with the normal sample database, so that potential pathogens should be identified based on their increased distribution compared to the normal human samples.

To facilitate the application of EOPM in multiple surveillance sites for infectious diseases, we designed software with a user-friendly interface, which is supported by a statistical analysis method based on a comprehensive microbial sequence identification database.

In microbial diagnostic microarrays, only a few probes are designed for each targeted microbe, and each probe should be confirmed with specific positive and negative samples. In the pan-microbial microarrays, many probes are designed for one pathogen, and there is no way to confirm each probe. However, the majority of the probes targeting an expected pathogen are likely to be positive, and not hybridize with other non-target microbes. We applied a hypergeometric distribution to calculate a p-value for each species as an assessment of statistical significance. Whether a pathogen was significantly present was determined using a complex interpretation method. The formula of hypergeometric distribution function is as follows:

$$p\left(i\geq m|N,M,n,m\right)=\sum_{i=m}^{n}\frac{C_{M}^{i}C_{N-M}^{n-i}}{C_{N}^{n}}$$

where *C* stands for the combination formula; *N* is the whole number of microbial probes on an array; *M* is the number of probes for a target microbe; *n* is the number of probes for which the intensity is positive on an array; and *m* is the number of probes whose intensity is positive for a target microbe. The probes were ranked by the signal of the Cy5 fluorescent dye that was used to label the patient sample. In the user-interface of the EOPM software, the proportion of probes can be chosen by the user according to the sample types. A small p-value indicates that there is a very low likelihood that a mistake has occurred in the multi-probe analysis, and correspondingly, that there is a high probability of the existence of the target microbe. Finally, the p-value is adjusted using Benjamini and Hochberg's FDR correction [15].

Because the probes were designed to both the species and genus levels, results will be given accordingly. In EOPM analysis, when there were at least three positive probes for a specific species of pathogen and an enrichment p-value < 0.01, the given species could be considered positive for further investigation, including the clinical symptom coincidence analysis.

### Sensitivity test for EPOM

Molecular detection methods, including pan-microbial microarrays and unbiased high throughput sequencing, traditionally rely on random amplification, and so have lower sensitivity than specific PCR [16]. Clinical samples usually contain host nucleic acid which may interfere with the sensitivity of microarray analysis. To determine the sensitivity of EPOM, we spiked viral RNA into human RNA, mimicking the actual clinical samples. Enterovirus 71 (EV71), a single-stranded RNA virus, was cultured with Vero cells. The RNA from the culture supernatant medium was extracted and quantitatively determined using a qRT-PCR standard curve. Then, 10^3^–10^8^ EV71 molecules were spiked into RNA extracted from 10^12^ human HeLa cells. The RNA was then randomly amplified and hybridized with the EOPM microarray as described above. In parallel, RT-PCR using a pair of specific primers to amplify EV71 was performed to compare the sensitivity of the two methods.

### EOPM verification using known pathogens and clinical sample tests

Known pathogens, including cell-cultured viral reference strains, cultured bacteria, and fungi, were used to verify EOPM performance. Clinical samples were all from patients with obvious infectious disease symptoms and which obtained negative results with routine diagnostic methods. Following detection by EOPM, the screened pathogens that caused similar clinical symptoms to those of the patients from which the clinical samples were collected were PCR amplified with species- or genus-specific primers. PCR-positive samples were then sequenced. This study obtained ethical approval from Ethical Committee of Guangdong Women and Children’s Hospital. Informed consent was not required because clinical samples were screened for potential pathogens *in vitro*. Original microarray data have been submitted to the Gene Expression Omnibus with the platform access number GPL16935.

## Results

### Evaluation of EOPM

High throughput microarrays with long oligonucleotide probes, such as the Virochip and GreeneChip systems, have proved effective for pathogen screening [9,11,17,18]. The EOPM technique described here also uses long oligonucleotide probes and random PCR amplification.

Several known viruses, bacteria, and fungi were used to evaluate the accuracy of EOPM. Dengue virus was used as a test subject to determine whether the EOPM method could detect the virus from an infected C6/36 cell culture (Tables 1, 2, and 3). As shown in Table 1, among the 15 top ranked probes, eight targeted dengue virus specifically, while a further four probes targeted related flavivirueses such as Phnom Penh bat virus, Tembusu virus, and deer tick virus. We also carried out enrichment analysis of the positive probes at both the species and genus level. Notably, only dengue virus or closely related species showed significant enrichment (Table 2), and only *Flavivirus* showed significant enrichment at the genus level (adjusted p-value<0.0001) (Table 3). Both results were consistent with the known cultured dengue virus.

**Table 1:** Top 15 probes identified in EOPM analysis of cell culture infected with dengue virus

**Probe**	**cy3 intensity**	**cy5 intensity**	**Ratio (cy5/cy3)**	**Species**	**Genus**
Vm.27	145	65529	452	*Dengue virus*	*Flavivirus*
Vm.20	218	65529	301	*Dengue virus*	*Flavivirus*
bacts.2149	287	65529	228	*Mycoplasma*	*Mycoplasma*
Vm.9835	232	52365	226	*Sendai virus*	*Respirovirus*
Vm.21	332	65529	197	*Dengue virus*	*Flavivirus*
Vm.23	334	65529	196	*Dengue virus*	*Flavivirus*
Vs1.7636	352	65529	186	*Phnom Penh bat virus*	*Flavivirus*
Vm.41	370	65529	177	*Dengue virus*	*Flavivirus*
Vm.9292	453	65529	145	*Tembusu virus*	*Flavivirus*
bacts.5220	286	37895	133	*Staphylococcus*	*Staphylococcus*
Vs1.7675	543	65529	121	*Deer tick virus*	*Flavivirus*
Vm.24	162	18867	116	*Dengue virus*	*Flavivirus*
Vm.1	280	31761	113	*Dengue virus*	*Flavivirus*
Vm.40	198	19474	99	*Dengue virus*	*Flavivirus*
Vs1.7671	791	65529	83	*Deer tick virus*	*Flavivirus*

The probes were ranked by ratio of cy5/cy3 intensity. Non-infected cell samples were labeled with cy3, and virus infected cell samples were labeled with cy5.

**Table 2:** **Enrichment analysis of pathogens at the species level in dengue virus**-**infected samples**

**Species**	**m**	**M**	**N-M**	**n**	**Adjusted p-value**
*Dengue virus*	14	41	55016	550	0.00E+00
*Deer tick virus*	5	10	55047	550	8.20E-06
*Mycoplasma*	4	55	55002	550	0.5073
*A*-*2 plaque virus*	2	8	55049	550	0.6017
*Phytomyza*	5	100	54957	550	0.6325
*Orf virus*	6	150	54907	550	0.6859
*Brevibacterium*	3	41	55016	550	0.9211

N: the total number of probes on the EOPM platform; M: the number of probes designed for a target species; n: the number of positive probes identified in a microarray; m: the number of positive probes for a species. The top 1% of the total number of probes was considered to be positive probes.

**Table 3:** **Enrichment analysis of pathogens at the genus level in dengue virus**-**infected samples**

**Genus**	**m**	**M**	**N-M**	**n**	**Adjusted p-value**
*Flavivirus*	42	1697	53360	550	1E-05
*Mycoplasma*	4	57	55000	550	0.3075
*Phytomyza*	5	97	54960	550	0.3321
*Mycoemilia*	3	29	55028	550	0.3609
*Aspergillus*	3	40	55017	550	0.6627
*Brevibacterium*	3	41	55016	550	0.6869
*Varicellovirus*	10	429	54628	550	0.7028
*Thogotovirus*	4	83	54974	550	0.7336
*Avipoxvirus*	7	272	54785	550	0.893228
*Orthopoxvirus*	10	496	54561	550	0.933598

N and n are the same as for Table 2. M: the number of probes designed for a target genus; m: the number of positive probes for a genus.

By following a similar procedure, we successfully tested EOPM on a panel of other known pathogens, including an RNA virus, a DNA virus, bacteria, fungi, and parasites (listed in Table 4).

**Table 4:** List of known pathogens from cultured samples or confirmed pathogens from clinical samples that were successfully detected by EOPM

**Species**	**Genus**	**Description**
*Sindbis virus*	*Alphavirus*	Positive stranded RNA virus
*Dengue virus*	*Flavivirus*	Positive stranded RNA virus
*Human immunodeficiency virus*	*Lentivirus*	Positive stranded RNA virus
*Entervirus 71*	*Enterovirus*	Positive stranded RNA virus
*Rubella virus*	*Rubivirus*	Positive stranded RNA virus
*Human parainfluenza virus*	*Respirovirus*	Negative stranded RNA virus
*Influenza B virus*	*Orthomyxovirus*	Negative stranded RNA virus
*Rotavirus A*	*Rotavirus*	Double stranded RNA
*Mammalian orthoreovirus*	*Orthoreovirus*	Double stranded RNA
*Human adenovirus*	*Mastadenovirus*	Double stranded DNA virus
*Group B streptococcus*	*Streptococus*	Gram positive bacteria
*Listeria monocytogenes*	*Listeria*	Gram positive bacteria
*Streptomyces cuspidosporus*	*Streptomyces*	Gram positive actinobacteria
*cryptococcus magnus*	*cryptococcus*	Fungi
*Toxoplasma gondii*	*Toxoplasma*	Parasite

In terms of detection sensitivity, EOPM could reliably detect EV71 when >10^6^ copies of EV71 RNA were mixed into 10^12^ copies of HeLa cell RNA, while 10^3^ copies of spike virus RNA could be detected in 10^12^ copies of host RNA by specific RT-PCR following agarose gel electrophoresis. Therefore, we inferred that when there was a high level of background nucleic acid, the detection sensitivity of random primer amplification was three orders of magnitude lower than specific primer amplification.

### Clinical case 1: identification of adenovirus responsible for an outbreak of flu-like infections

**Figure 1:**
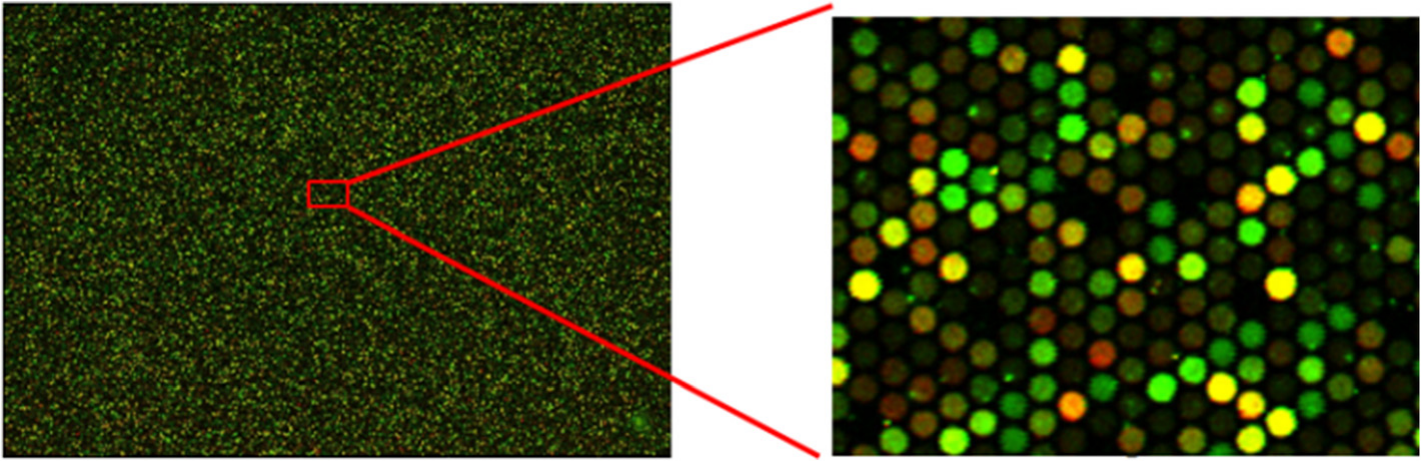
**Hybridization picture of EOPM in the adenovirus outbreak case.** RNA/DNA from patients was labeled with red cy5 fluorescent dye, and nucleic acid from normal control serum was labeled with green cy3 dye.

Most adenovirus infections cause similar symptoms to those induced by some respiratory viruses and mycoplasmas, making it difficult to identify the pathogens by traditional clinical diagnostic procedures. In February of 2012, an outbreak of disease caused by an unknown pathogen occurred in Baoding City, Hebei Province. Patients presented with obvious infectious symptoms, such as high fever, coughing, throat congestion, lung tissue necrosis, and bronchopneumonia. Initially, influenza virus, SARS virus, and mycoplasma, known causes of these clinical symptoms, were suspected, but PCR tests were negative for all three pathogens. To rapidly identify the unknown pathogen, EOPM chips were selected to screen the possible pathogens responsible for these infections. Nucleic acid was extracted from patient serum samples to be used for EOPM analysis. Nucleic acid from normal serum was used as a control. One scanned microarray image is shown in Figure 1, and the enrichment results for the top-ranked pathogens at species and genus level are listed in Tables 5 and 6 respectively. Adenoviruses were found to be significantly enriched, as were the top five species results (Tables 5 and 6). We further verified adenovirus as the causative agent by PCR targeted to a conserved region of *Mastadenovirus* genomic sequence (see Additional file 1).

**Table 5:** Enrichment result at the species level for EOPM analysis of the clinical outbreak case of respiratory infection

**Species**	**m**	**M**	**N-M**	**n**	**Adjusted p-value**
*Unclassified adenovirus*	14	150	54907	550	3.28E-08
*Human adenovirus*	11	80	54977	550	3.94E-08
*Human adenovirus*_*type 35*	5	12	55045	550	5.99E-06
*Human adenovirus type 34*	4	6	55051	550	1.20E-05
*Human adenovirus type 7*	4	22	55035	550	0.0049
*Streptomyces*	9	256	54801	550	0.0726

**Table 6:** Enrichment result at the genus level for EOPM analysis of the clinical outbreak case of respiratory infection

**Genus**	**m**	**M**	**N-M**	**n**	**Adjusted p-value**
*Mastadenovirus*	54	455	54602	550	0.00E+00
*Entomophthora*	4	12	55045	550	0.0003
*Streptomyces*	9	258	54799	550	0.0728
*Parvovirus*	11	439	54618	550	0.2336
*Erythrovirus*	7	204	54853	550	0.2451
*Streptococcus*	5	118	54939	550	0.3556
*Enterovirus*	10	1964	53093	550	0.3957

### Clinical case 2: cardiovirus discovery in a hand-foot-and-mouth juvenile patient

Hand-foot-and-mouth disease (HFMD) is a common viral illness that predominantly affects infants and children younger than 5 years old. HFMD epidemics usually occur in China in late spring and early summer. The pathogens responsible for HFMD are mainly coxsackie A16 virus (CVA16) and enterovirus 71 (EV71), both of which belong to the *Enterovirus* genus. The routine HFMD clinical diagnosis includes three qRT-PCR kits targeting the *Enterovirus* genus, CVA16, and EV71 species respectively.

In May of 2010, many children were found to have clinical symptoms of “hand-foot-and-mouth diseases” at Guangdong Women and Children’s Hospital, located in southern China. Although most patients were diagnosed as having CVA16 or EV71 infections by the qRT-PCR analysis, some were negative for *Enterovirus*. To identify the pathogens responsible for *Enterovirus*-negative HFMD children, samples from each of the patients were subjected to EOPM analysis. About 1 mg of a feces sample was used to extract RNA, using a TIANamp Virus DNA/RNA Kit, and labeled with Cy5 following random amplification. In parallel, RNA extracted from normal feces was labeled with Cy3 and used as a control. The enrichment analysis at the species level identified Theiler’s-like *Cardiovirus* as the most probable pathogen responsible for the HFMD infection in these patients (Table 7). Analysis of the enrichment results at the genus level revealed *Cardiovirus* as the number one match, showing significant enrichment (Table 8). The genera *Cardiovirus* and *Enterovirus* belong to the family *Picornaviridae*, a family of positive single-stranded RNA viruses. A few intestinal viruses of the Picornaviridae family, besides the enterviruse strains coxsackie A virus and enterovirus 71, are also known to potentially cause HFMD syndrome. Therefore, we hypothesized that the *Enterovirus*-negative HFMD children were actually infected with *Cardiovirus*, the sister genus of *Enterovirus*. To confirm the presence of *Cardiovirus* in patent feces, two specific nested RT-PCR primers proposed in a previous report [19] were used to amplify the RNA extracted from the *Enterovirus*-negative patients. Samples were *Cardiovirus*-positive (see Additional file 2). The PCR products were further verified by DNA sequencing, and 708 bp of the PCR amplicon shared 99% nucleotide identity with human TMEV-like *Cardiovirus* isolate UC2 5' UTR.

**Table 7:** **Enrichment result at the species level for EOPM analysis of the *Enterovirus***-**negative HFMD patients**

**Species**	**m**	**M**	**N-M**	**n**	**Adjusted p-value**
*Theiler*-*like virus NGS910*	5	10	55047	550	1.19E-06
*Theiler*'*s encephalomyelitis virus*	5	30	55027	550	0.0006
*Sleeping disease virus*	2	11	55046	550	0.2259
*Avian sarcoma virus*	2	24	55033	550	0.6795
*Congo hemorrhagic fever virus*	2	54	55003	550	0.9891
*Chagres virus*	1	10	55047	550	0.9924

**Table 8:** **Enrichment result at the genus level for EOPM analysis of the *Enterovirus***-**negative HFMD patients**

**Genus**	**m**	**M**	**N-M**	**N**	**Adjusted p-value**
*Cardiovirus*	15	110	54947	550	6.42E-12
*Muromegalovirus*	3	190	54867	550	0.9594
*Coemansia*	2	96	54961	550	0.9637
*Arthrobacter*	2	108	54949	550	0.9771
*Nairovirus*	2	146	54911	550	0.9923

The microarray raw data of other symptom-causing pathogens, such as streptococcus and mycoplasma, identified by EOPM in peripheral blood in infectious patients, were also submitted to the GEO database.

### Development of software with a user-friendly interface to support the EOPM application

**Figure 2:**
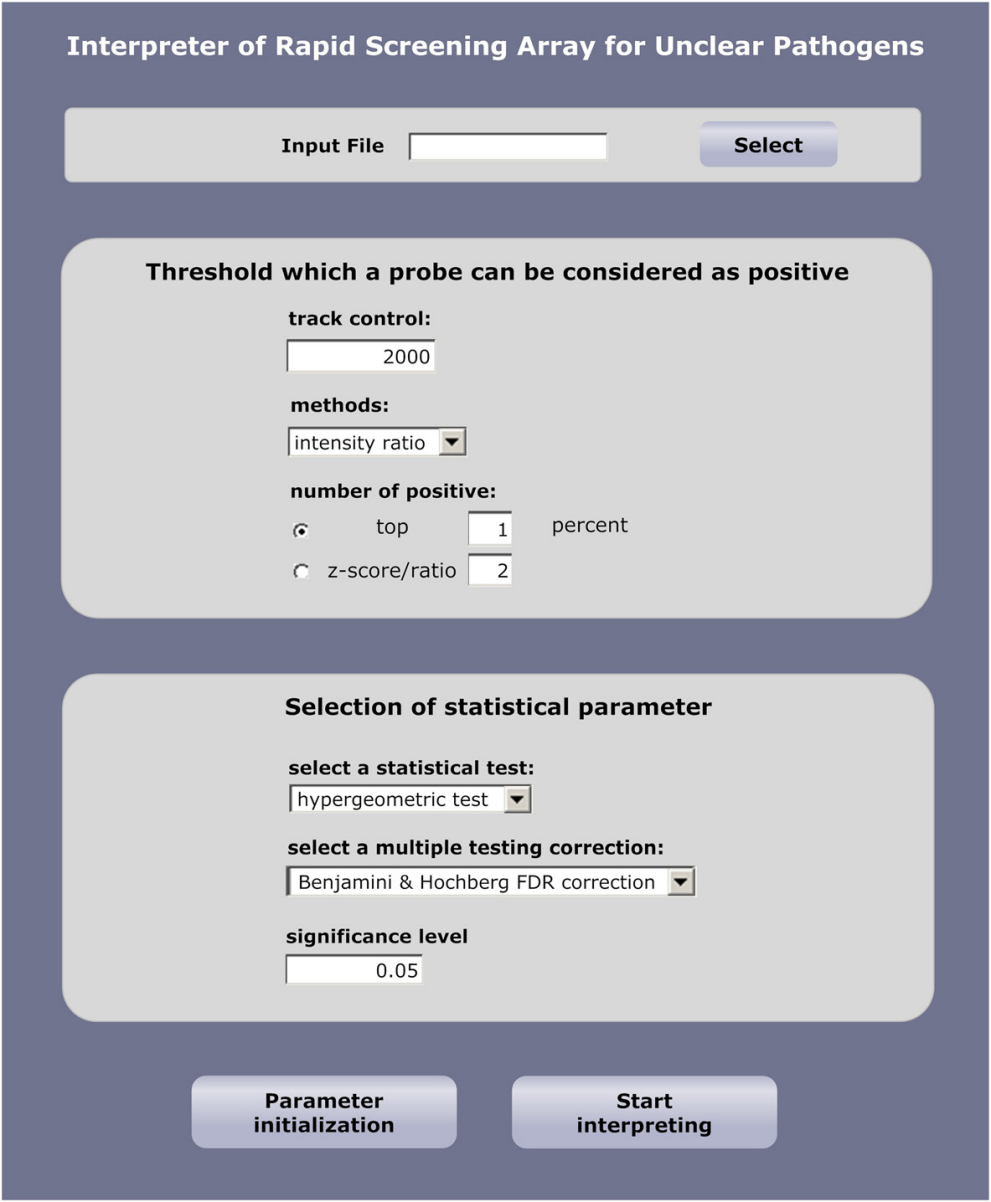
**User interface of the EOPM software.** Users will generally only need to input raw microarray data for pathogen identification.

The primary purpose of developing the EOPM was to facilitate the rapid identification of unknown pathogens in regional surveillance centers in China when emergent pathogen-causing incidents occur. When considering the application of microarray technology, data analysis is a significant obstacle to users without specialized knowledge in bioinformatics analysis of microarray data and nucleic acid sequences. Therefore, we implemented the statistical enrichment analysis in a user-friendly interface (Figure 2). The software can support a large-scale search of probe hits against a comprehensive microbial sequence database. We believe this software will greatly facilitate the installation of the EOPM platform in different infectious surveillance system laboratories in China. The software can be accessed at http://www.genestone.com.cn:8080/microbial/index.jsp.

## Discussion

Since the first application of a high-throughput, rapid, and unbiased microarray for detecting viral pathogens in 2002 [7], several pan-microbial microarray platforms with different degrees of coverage of various pathogens have been established. These microarray platforms use long oligonucleotide probes (60–70-mer) and random PCR amplification, and have successfully identified unexpected pathogens in infectious disease outbreaks, even discovering novel viruses with homology to known species [8,11]. In this study, we constructed a high-density EOPM array for screening all known viruses, bacteria, fungi, and parasites that could become vertebrate pathogens. Based on the sequence data available for vertebrate pathogens, we have designed 60,000 60-mer oligonucleotide probes targeting 2,554 vertebrate virus species (involving 151 genera, 36 families), 124 bacterial genera (involving 53 families), 38 fungal genera (involving 17 families), and 47 parasite genera (involving 24 families). The 60-mer oligonucleotide probes can cross-hybridize with similar but non-identical sequences, allowing the detection of novel pathogens that are related to known species. The EOPM probes designed to detect bacteria, fungi, and parasites were targeted to 16S rRNA or 18S rRNA sequences. Whereas rRNA sequences are relatively conserved in the same genus or family, EOPM can distinguish bacteria, fungi, and parasites at either the genus or family level, which has already been successfully applied in a clinical setting for confirmation and treatment. In the sensitivity study of EOPM, we designed experiments to compare the sensitivity of random amplification and specific amplification, while not considering the effect of other issues, such as clinical sample collection and nucleic acid extraction, on the sensitivity of EOPM. EOPM showed 10^3^-fold lower sensitivity than specific target PCR amplification, which was consistent with a previous report [20]. The lower sensitivity was due to the random PCR amplification adopted in the EOPM sample preparation, which was not as efficient as specific PCR for amplification of a particular species. Despite having lower sensitivity than target-specific PCR, the EOPM platform is sufficiently sensitive to identify the pathogens causing clinical symptoms in infectious outbreaks, in which symptom-causing pathogens should be highly enriched. The sensitivity can be further improved in practice if acellular samples with minimal host nucleic acid contamination, such as serum and throat swabs, are used for pathogen screening. For example, Greninger and colleagues had used ViroChip microarray to identify influenza A/H1N1 in nasal swab samples showing a comparable sensitivity with RT-PCR [10]. In the sample preparation for the EOPM method, all RNA and DNA extracted from samples are firstly reverse transcribed. RNA viruses are converted into cDNA, and DNA viruses keep its DNA status in the reverse transcription reaction, then the DNA, including the reverse-transcribed cDNA and original DNA viruses, were transformed to double strand DNA for the subsequent random amplification procedure. Therefore, EOPM can detect both RNA viruses and DNA viruses in the same standard protocol. For bacteria, fungi, and parasites, EOPM detects 16S rRNA or 18S rRNA copies encoded by rRNA genes located in the genomic DNA. Because rRNA genes are highly transcribed, detecting rRNA molecules instead of rRNA genes should achieve higher sensitivity.

With the dual color strategy used by the EOPM method, one normal sample without infectious symptoms was always analyzed in parallel. Despite this, the “normal” sample may possess its own clinical characteristics. For example, we have found Torque teno virus and human endogenous retroviruses in some normal blood samples. These viruses do not cause obvious clinical symptoms, and should not interfere with the aim of EOPM analysis, which is to determine the possible pathogens causing the symptoms in the test patients. EOPM data analysis consists of two steps. First, we screened for significantly enriched microbes in the target sample compared with the normal sample using the dual color chip. Second, the predicted microbes identified in the first step were compared with a database compiled from the normal population mentioned above, to eliminate the background microbes that also exist in normal samples without infectious symptoms.

Pan-microbial screening microarrays differ from nucleic acid-based microbial diagnostic technologies, such as qPCR and low density microarrays. These diagnostic technologies are merely aimed at identifying one or a few types of microbes using target-specific probes that should be confirmed with specific positive and non-specific samples. Moreover, diagnostic low-density microarrays usually use short oligonucleotides of about 20-nucleotides as specific probes, similar to TaqMan probes in qPCR technology [21,22]. The very limited number of short probes/primers targeting a pathogen could fail to detect sequences with mutations located in the regions targeted by the probes/primers. However, over a dozen long oligonucleotide probes were designed for each pathogen in the EOPM method, allowing reliable identification of a pathogen based on a statistical enrichment analysis of the probe group, instead of one individual probe. Moreover, EOPM can effectively narrow down the potential pathogens and even identify novel pathogens in complex clinical infection situations.

In addition to the pathogen sequences, 1160 host immune response genes were also included in the EOPM database. During EOPM analysis of clinical samples, the immune response genes show dramatic up- or down-regulation in the target samples compared with the normal reference (data not shown). So far we have not found any reliable relationships between the immune response genes and the pathogen categories. The overall clinical information for patients and normal controls should also be comprehensively analyzed. Human immune related genes in peripheral blood show dramatic differences in expression even in a normal population, with differences correlated with sex, age, and sampling time, amongst other factors [23,24].

Until now, the available genome-wide technologies to detect unknown pathogens in infectious outbreaks primarily consisted of microarrays and NGS. Although NGS can provide the most in-depth, unbiased information, and can reveal completely novel pathogens, it is time-consuming when the sample contains hundreds of microbial species that require comprehensive data processing. Therefore, NGS cannot meet the short time requirement for infectious disease control. However, the most complicated step in EOPM technology is probe design, which can be undertaken by a core bioinformatics team in the development phase. Once probe design is complete, and the whole microarray procedure is optimized as a standard procedure, pathogen screening results can be interpreted in less than 28 hours. Therefore, EOPM is more suitable for applications requiring detection of unknown pathogens during infectious outbreaks.

In addition, with the rapid increase in microbial metagenomic sequence data produced by NGS, the probes used for EOPM can easily be upgraded, and the EOPM version can be updated due to the *in situ* synthesis technology replacing the spotting technology in microarray fabrication.

## Conclusions

In conclusion, EOPM is a very powerful pan-microbial detection microarray platform, which can detect almost all known pathogens and related species. In several clinical test applications, we found that EOPM technology is sensitive enough to detect the pathogens causing evident clinical symptoms. EOPM is designed for easy operation, with detection software containing a user-friendly interface, facilitating its application in molecular laboratories. Infectious disease epidemics emerge frequently in China, and we believe that the use of EOPM in main pathogen surveillance sites across the country could play an important role in infectious disease control in China.

## Competing interests

There are patents pending by the authors related to the probe design methods and array data statistical enrichment methods. In addition, software copyright is pending related to pathogen interpretation.

## Authors’ contributions

LZ and YJ conceived the study and analyzed the data. LZ drafted the manuscript. WH and YY conducted the microarray experiments, PCR, and sequencing confirmation. XZ and HL designed probes and software. XZ, AY, CZ, and ZH participated in the sample collection and array data analysis. All authors read and approved the final manuscript.

## Pre-publication history

The pre-publication history for this paper can be accessed here:

http://www.biomedcentral.com/1471-2334/13/437/prepub

## Supplementary Material

Additional file 1 Two pairs of specific primers for amplifying adenovirus, and the sequence of PCR products from clinical case 1. Click here for file

Additional file 2 Sequence of nested RT-PCR primers for cardiovirus, and the PCR product sequence from clinical case 2. Click here for file
