# Supplementary material for: An easy operating pathogen microarray (EOPM) platform for rapid screening of vertebrate pathogens
Source: BMC Infect Dis. 2013 Sep 20;13:437. doi: 10.1186/1471-2334-13-437 (PMC3848773; doi:10.1186/1471-2334-13-437)
Supplement: Additional file 1 — Two pairs of specific primers for amplifying adenovirus, and the sequence of PCR products from clinical case 1. [file 1471-2334-13-437-S1.docx]

Additional material

Additional file 1: The two pair of specific primers for amplifying adenovirus and the sequence of PCR products in the clinical case 1

Primers 1:

Forward primer: 5’-GCAGTGGTCGTACATGCACAT-3’;

Reverse primer: 5’- CACGGTGGGGTTTCGAAACTTG-3’;

PCR product length: ～127bp

PCR product sequence:

5’-TCTGAGTCGGGTCTGGTGCAGTTCGCCCGCGCCACAGACACCTACTTCAATCTGGGAAATAAGTTTAGAAATCCCACCGTAGCGCCGACCCACGATGTGACCACCGATCGTGACTGGGAAACAAGTTTCGAAACCCCACCGTGA-3’

Primers 2:

Forward primer: 5’-GCAGTGGTCGTACATGCACAT-3’;

Reverse primer: 5’- CCATGTCCAGCACTCGGTTGTC-3’,

PCR product length: ～266 bp

PCR product sequence:

5’-AGAGTCGGGTCTGGTGCAGTTCGCCCGCGCCACAGACACCTACTTCAATCTGGGAAATAAGTTTAGAAATCCCACCGTAGCGCCGACCCACGATGTGACCACCGATCGTAGCCAGCGGCTCATGTTGCGCTTCGTGCCCGTTGACCGGGAGGACAATACATACTCTTACAAAGTGCGGTACACACTGGCCGTGGGCGACAACCGAGTCCTGGACATGGA-3’
