# Supplementary material for: An easy operating pathogen microarray (EOPM) platform for rapid screening of vertebrate pathogens
Source: BMC Infect Dis. 2013 Sep 20;13:437. doi: 10.1186/1471-2334-13-437 (PMC3848773; doi:10.1186/1471-2334-13-437)
Supplement: Additional file 2 — Sequence of nested RT-PCR primers for cardiovirus, and the PCR product sequence from clinical case 2. [file 1471-2334-13-437-S2.docx]

Additional material

Additional file 2: the sequence of Nested RT-PCR primers for cardiovirus in the clinical case 2 and the PCR product sequence:

Additional table 1. The sequences of nested RT-PCR primers for confirm cardiovirus in the Enterovirus-negative HFMD patient

| Primers (Nested RT-PCR) | sequence (5’-3’) | Position | oriented |
| --- | --- | --- | --- |
| First round | CTAATCAGAGGAAAGTCAGCAT | 188-209 | + |
| Second round | CAGCATTTTCCGGCCCAGGCTAA | 204-226 | + |
| Second round | GCTATTGTGAGGTCGCTACAGCTGT | 718-742 | - |
| First round | GACCACTTGGTTTGGAGAAGCT | 990-1011 | - |

PCR product sequence:

5’-TAGATTTGATTTAATCCCTTGACGAATTCGGAATGAGATGTTCTCCCTCCCTTGCCGCTTGTTCACACCCACTGTTTTGATTCGGCCCCTTGTGACAAGCCCCTTGGTGAAAGAACCTCTCTCTTTTCGACGTGGTTGGAATTAACATCACTTCCGACGAAAGTGCTATCATGCCTCCCCGATTATGTGATGTTTTCTGCCCTGCTGGGCGGAGCATTCTCGGGTTGAGAAACTTTGACTCTTTTCTTTTGGAACCTTGGTTCCCCCGGTCTAAGCCGCTTGGAATATGACAGGGTTATTTTCCTTATTCTTGATTCTACTTTTATGGGTTCTATCCATAAAAAGGGTACGTGCTGCCCCTTCCTTCTTTGGAGAATTCACACGACGGTCTTTCCGTCTCTCAACAAGTGTGAATGCAGCATGCCGGAAACGGTGAAGAAAACAGTTTTCTGTGGAAGTCTAGAGTGCACATCGAAACAGCTGTAGCGCCCC-3’
